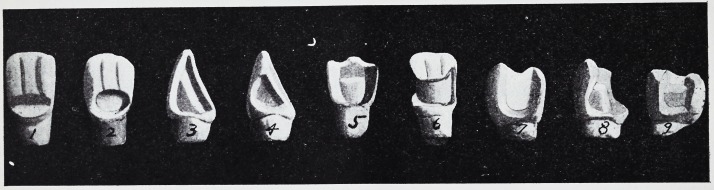# Preparation of Cavities for Porcelain Inlays

**Published:** 1904-07

**Authors:** Frank E. Cheeseman

**Affiliations:** Chicago.


THE AMERICAN

JOURNAL OF DENTAL SCIENCE

### PREPARATION OF CAVITIES FOR PORCELAIN INLAYS.

By Frank E. Cheeseman, D. D. S., Chicago.

(Written for *The American Journal of Dental Science.*)

The preparation of cavities for porcelain inlays should not differ materially from the method used for gold fillings as far as retention is concerned, with the exception that there must be no undercuts or interlocking and the cavity must be so shaped that the matrix will draw from it without distortion after having been swaged or burnished.
served and one that invites failure if neglected is that the cavity must be so prepared that when completed the inlay shall have a definite seat which should be as broad as the base as is possible without endangering the pulp.

This to some may seem to be a radical statement, but experience will teach that we cannot hope to do successful inlay operations unless we obtain all the mechanical retention possible.

The walls of the cavity should be nearly parallel and at right angles with the pulpal wall, with a slight divergence outward until the periphery is reached.

This will result in a cavity with narrowest point at the base, which will admit of drawing of the matrix and also will give a perfect adaptation to all cavity surfaces. The margins must be as smooth and sharp as possible, so that there shall be no frail edges of porcelain, and should extend in an unbroken line around the entire cavity. This will give a sharp outline to the matrix, which is indispensable to perfect adaptation.

Gem stones should be used whenever possible as they greatly facilitate the operation in that they cut with more accuracy and rapidity than does the bur.

The Arkansas stones should be used for polishing margins, as their use results in a smooth, sharp surface. They must be used carefully, keeping them wet and revolving engine at high speed. Avoid much pressure as they are delicate and easily broken.

In the class of cavities where these stones cannot be used it will be necessary to resort to burs, polishing margins with disks and dull gold finishing burs.

The rubber dam should be used almost invariably, as more thorough preparation and satisfactory results will follow its use. However, care must be taken that shade for inlay should be selected before its adjustment, as the drying of the tooth will cause temporary change of color.

The saucer-shaped preparation of cavities should never be used. The term is misleading to the inexperienced worker and if taken literally by him must result in discouraging failures, with a loss of confidence in the value of inlay operations due to this unscientific method of preparation.

At the present time there is no cement upon which we can depend for retention of an inlay, unless some form of mechanical retention has been obtained in the preparation of the cavity.

Taken literally a labial cavity, using saucer-shaped preparation, would result as represented in the following illustration, Fig. 1. Although the inlay would be subjected to no stress, still it would be folly to place it in this cavity, expecting it to stay in place. There being no mechanical retention we should be obliged to depend upon the adhesiveness of the cement to both tooth and inlay for retention which, in the experience of the writer, has always proved to be a failure.

Preparation for this class of cavities should be made as follows. (Fig. 2.) Here the floor of the cavity is flat, which serves as a seat, the walls nearly parallel with a slight divergence outward, which will admit of removal of matrix without distortion and for perfect contact of completed inlay to all cavity surfaces.

For a cavity prepared like this the cement simply acts as an adhesive medium between the inlay and tooth, the mechanical retention having been secured in the preparation of the cavity.

In the preparation of proximo occlusal cavities for incisors (Fig. 3) the floor should be extended lingually to admit of the widest possible seat, with a slight groove cervically, lingually, and at the incisal edge for retention. For pulpless teeth this preparation may be extended with better probability of success, as the broader the seat, the better opportunity we have for successful retention of inlay.

In the preparation of simple approximal cavities for incisors and cuspids the walls should be nearly parallel and at right angles with the pulpal wall. (Fig. 4.)

Much has been said as to the inherent edge weakness of porcelain, and certain writers and those joining in the discussion have on that score condemned its use for compound occlusal cavities in bicuspids and molars. This argument was intended as a warning to the inexperienced rather than a belief that porcelain inlays or fillings are not applicable for this class of cavities, at least it has been so admitted by one of these gentlemen. There is no question that it would be much safer to limit its use to the anterior teeth until experience has made the operator proficient in the perfect fitting of matrices and in the proper manipulation of the porcelain.

After that point has been reached it is only a matter of judgment of the operator as to its use in all classes of cavities, except fissure cavities in bicuspids and molars and pit cavities on the lingual aspect of the anterior teeth where gold foil fillings are clearly indicated.

The two following methods of preparation will safe-guard the margins of the inlay:

First, by extending the margins of the cavity beyond any point of contact with occluding tooth; second, if such procedure will result in the unnecessary loss of tooth structure by grinding the occluding tooth at the point it would come in contact with margins of inlay.

When such extension is resorted to care must be taken that allowance be made for a sufficient bulk of porcelain to fully withstand the stress of mastication.

These safeguards taken, there is much less liability to the fracture of porcelain than there is to a recurrence of decay at the cervical margins of gold fillings, and the consequences are much less grave if it should take place, the defective point being on a self-cleansing surface and under easy observation of the dentist.

For this class of cavities, which are generally extensive, it is good practice to make use of the step formation wherever possible, as they are more self-retentive, using the same general plan of walls, diverging slightly from the parallel with a broad seat for retention, keeping in mind that cavity must be deep enough that exposed point of inlay shall have sufficient bulk of porcelain to withstand stress of mastication.

The following illustrations are as nearly as possible reproductions of formation of cavities that have been used in practical cases by the writer. Fig. 5 represents a central incisor, involving one-third of the incisal edge. The entire lingual portion of the tooth was cut to the gingival margin to allow of a broad seat for retention, with grooves mesially and distally, also using the step formation. For this class of cavities extensive preparation is necessary to obtain true mechanical retention.

Fig. 6 represents a mesio labia distal cavity in a central incisor, which when presented had defective gold fillings "which had been inserted six months previously by a good operator" in the mesial and distal surfaces and two cement fillings on the labial surface teeth were very defective in structure, presenting what seemed to be a hopeless condition and the dental organs presenting a discouraging appearance. Removed all the fillings and made one compound cavity extending well into sound territory. This inlay has rendered good service for eighteen months, with no recurrence of decay.

Fig. 7 represents a common form of bicuspid restoration. The formation of the cavity is such as not to admit of the step preparation.

Fig. 8 represents a more complicated cavity in bicuspid, necessitating the restoration of the lingual cusp.

Fig. 9 a proximo occlusal cavity in molar, using the step method of preparation.

This paper has been prepared with a view of encouraging those about to take up this class of work, and if possible to restimulate the interest of those who have abandoned its use as a result of unscientific cavity preparation. I have endeavored to bring out clearly and concisely the point which I consider necessary to successful cavity preparation, and if I have sufficiently urged the necessity for the maximum amount of mechanical retention possible to obtain and have made clear and emphatic enough that no dependence can be placed upon cement for this purpose and that it simply acts as an adhesive medium between inlay and tooth, my time will not have been spent in vain.

---

### MAKING THE MATRIX.

By Lee K. Stewart, D. D. S., Chicago.

(Written for *The American Journal of Dental Science.*)

## Figures and Tables

**Figure f1:**